# Artificial Intelligence–Enabled Facial Privacy Protection for Ocular Diagnosis: Development and Validation Study

**DOI:** 10.2196/66873

**Published:** 2025-07-09

**Authors:** Haizhu Tan, Hongyu Chen, Zhenmao Wang, Mingguang He, Chiyu Wei, Lei Sun, Xueqin Wang, Danli Shi, Chengcheng Huang, Anping Guo

**Affiliations:** 1 Department of Preventive Medicine, Shantou University Medical College, 22 Xinling Rd, Shantou, 515031, China, 86 13318055534; 2 Department of Optoelectronic Information Science and Engineering, Physical and Materials Science College, Guangzhou University, Guangzhou, China; 3 Han’s Laser Technology Industry Group Co., Ltd, Shenzhen, China; 4 Joint Shantou International Eye Center of Shantou University and The Chinese University of Hong Kong, Shantou, China (Hong Kong); 5 The Hong Kong Polytechnic University, Kowloon, Hong Kong, China (Hong Kong); 6 Department of Ophthalmology, the Fourth Affiliated Hospital of Harbin Medical University, Harbin, China; 7 University of Science and Technology of China, Hefei, China; 8 Department of Pharmacy, First Affiliated Hospital of University of Science and Technology of China, Division of Life Sciences and Medicine, University of Science and Technology of China, Hefei, China

**Keywords:** facial biometric data, Digital FaceDefender, privacy protection, auxiliary diagnosis, artificial intelligence, ocular disease

## Abstract

**Background:**

Facial biometric data, while valuable for clinical applications, poses substantial privacy and security risks.

**Objective:**

This paper aims to address the privacy and security concerns related to facial biometric data and support auxiliary diagnoses, in pursuit of which we developed Digital FaceDefender, an artificial intelligence–driven privacy safeguard solution.

**Methods:**

We constructed a diverse set of digitally synthesized Asian face avatars representing both sexes, spanning ages 5 to 85 years in 10-year increments, using 70,000 facial images and 13,061 Asian face images. Landmark data were separately extracted from both patient and avatar images. Affine transformations ensured spatial alignment, followed by color correction and Gaussian blur to enhance fusion quality. For auxiliary diagnosis, we established 95% CIs for pixel distances within the eye region on a cohort of 1163 individuals, serving as diagnostic benchmarks. Reidentification risk was assessed using the ArcFace model, applied to 2500 images reconstructed via Detailed Expression Capture and Animation (DECA). Finally, Cohen Kappa analyses (n=114) was applied to assess agreement between diagnostic benchmarks and ophthalmologists’ evaluations.

**Results:**

Compared to the DM method, Digital FaceDefender significantly reduced facial similarity scores (FDface vs raw images: 0.31; FLAME_FDface vs raw images: 0.09) and achieved zero Rank-1 accuracy in Pose #2-#3 and Pose #2-#5, with near-zero accuracy in Pose #4 (0.02) and Pose #5 (0.04), confirming lower reidentification risk. Cohen Kappa analysis demonstrated moderate agreement between our benchmarks and ophthalmologists’ assessments for the left eye (κ=0.37) and right eye (κ=0.45; both *P*<.001), validating diagnostic reliability of the benchmarks. Furthermore, the user-friendly Digital FaceDefender platform has been established and is readily accessible for use.

**Conclusions:**

In summary, Digital FaceDefender effectively balances privacy protection and diagnostic use.

## Introduction

With the rapid advancement of artificial intelligence (AI) applications in medical imaging, a vast array of newly generated medical images now encompass a wide range of personal information, including nonbiometric, physiological, behavioral biometric, and soft biometric identifiers [1]. These images undergo digitization, storage, transmission, and retrieval by healthcare organizations for various purposes [2,3]. However, this digitalization process has brought about significant concerns regarding security and multifaceted privacy, spanning these identifiers and more. In response to these concerns, the primary objective of our study is to develop a model that effectively extracts critical features for auxiliary diagnosis in eye hospitals while simultaneously ensuring patient privacy protection.

In their seminal work published in Nature Medicine, Yang et al [4] introduced the concept of a “Digital Mask” (DM) designed to protect patient privacy while preserving disease-relevant features critical for diagnosis. While this innovation represents a significant step forward in privacy preservation, Meeus et al [5] subsequently raised concerns about the reidentification risks associated with the DM. Specifically, their study extracted frames from facial videos provided by Yang et al [4], with one frame serving as a reference image and another used to compute the mask. Facial regions, excluding the eyes, were masked while maintaining facial contours, thus creating the “DMface.” The Faces Learned with an Articulated Model and Expressions (FLAME) model was then applied to generate a novel facial mask, termed “FLAME_DMface,” by integrating the Skinned Multi-Person Linear Model body model [5-7]. Meeus et al [5] evaluated the reidentification risks by comparing FLAME_DMface with the corresponding reference images using the Additive Angular Margin Loss-based ArcFace model. Their findings revealed potential reidentification vulnerabilities [5,8].

Yang et al [9] responded by emphasizing the secure maintenance of the original clinical examination videos, arguing that reidentification attacks using the FLAME model would be rendered irrelevant if the original videos were kept inaccessible. While we acknowledge the efforts of Yang et al [9] to address patient privacy concerns and mitigate identification risks, we also concur with the feedings by Meeus et al [5] that the reidentification risks associated with DM persist following FLAME processing. The retention of facial contours, including key regions such as the face, nose, eyes, and mouth, underscores the necessity for refining deidentification techniques to further reduce the risk of unauthorized reidentification.

In clinical environments, the balance between maintaining patient privacy and enabling auxiliary diagnosis is of paramount importance, particularly in specialized fields like ocular disease. In such cases, partial exposure of the eyes and periocular region may be necessary, but DM’s focus on extracting disease-relevant features from a limited eye region—comprising only the upper and lower eyelids and the iris—may be insufficient for comprehensive disease characterization, as shown in Multimedia Appendix 1.

Furthermore, previous research, such as that by Neumann et al [10], has highlighted the negative impact of extensive facial coverings in medical settings. Such coverings may hinder clinician-patient communication, diminish empathy [11], and reduce diagnostic accuracy [12] by obscuring essential facial cues [13] related to age, sex, and expression. These challenges became particularly evident during the COVID-19 pandemic and among Muslim women wearing veils [14-16]. As illustrated in Multimedia Appendix 1, DM may exacerbate these issues due to its extensive coverage, which is visually comparable to a white plaster cast.

In our study, we first developed an AI-driven method—Digital FaceDefender—and assessed the reidentification risks and the mean similarity between images generated using our proposed method and the DM approach. In addition, we developed and validated an auxiliary diagnostic benchmark. We anticipate that this innovative approach will enhance the diagnostic capabilities of medical professionals, providing essential support for early-stage patient evaluation while ensuring robust patient privacy protection.

## Methods

### Overview

**Figure 1.**
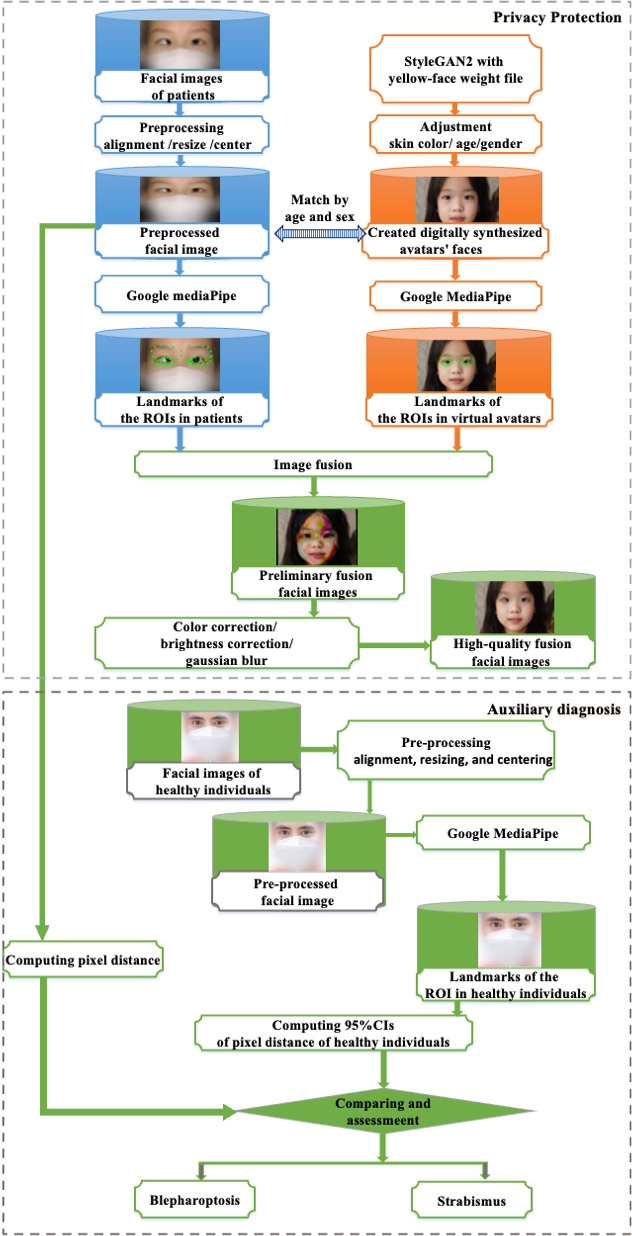
The workflow of Digital FaceDefender. ROI: region of interest.

In this study, we introduced Digital FaceDefender, a method designed to enhance privacy protection while supporting auxiliary diagnosis of ocular diseases in eye hospitals. Figure 1 shows the detailed workflow, with the region of interest (ROI) clearly marked.

### Ethical Considerations

The research protocol was reviewed and approved by the Institutional Review Board/Ethics Committee of the Fourth Affiliated Hospital of Harbin Medical University (approval 2023-Ethics Review-54; see Multimedia Appendix 2). All participants provided informed consent in accordance with the principles outlined in the Declaration of Helsinki.

To protect privacy, all face images presented in the paper were generated using our image fusion method or masked rather than displaying original face images from our private database.

### Dataset

In this retrospective study, we collected face images from 3 sources: 2 publicly available datasets (Flickr-Faces-HQ [FFHQ] and CASIA-FaceV5) and 1 private dataset from the Fourth Affiliated Hospital of Harbin Medical University. The images from the latter dataset were randomly selected.

Based on their extensive clinical diagnostic experience, two board-certified ophthalmologists (one Chief Physician and one Associate Chief Physician), both licensed by the Ministry of Health of the People’s Republic of China, independently evaluated a random sample of facial images from 1136 healthy individuals in our private database. These ophthalmologists, affiliated with the Joint Shantou International Eye Center and the Department of Ophthalmology at the Fourth Affiliated Hospital of Harbin Medical University, assessed the images using a standardized diagnostic protocol. Only individuals exhibiting clear ocular abnormalities, including esotropia, exotropia, vertical strabismus, and ptosis, were further assessed and included in this study. To ensure diagnostic consistency, the evaluations were performed independently, and inter-rater agreement was quantified using the Cohen Kappa coefficient.

### Image Preprocessing

Several preprocessing techniques were used to ensure the consistency and quality of patient images. Initially, facial alignment was performed by rotating the original images based on the line connecting the irises, ensuring the face was centered and horizontally oriented. All images were resized to 1024×1024 RGB (red, green, and blue) color space, the standard required for generating digitally synthesized avatars. If the original image size was smaller than this resolution, a white background was added; if larger, the image was cropped to maintain central facial positioning. Given that patient images are captured under variable conditions, including differences in lighting and background noise, a standard RGB color correction method [17,18] was used to adjust brightness, ensuring consistency with the digitally synthesized avatars’ faces.

### Creation of Digitally Synthesized Avatars

The FFHQ dataset, consisting of 70,000 high-quality PNG images with a resolution of 1024×1024 pixels, was used to the style-based generative adversarial network architecture (StyleGAN2) [19] model, which is known for generating realistic digitally synthesized avatars. However, this dataset is biased towards Caucasian faces (69% Caucasian, 4% Black, and 27% other) raising concerns regarding generalizability to more diverse populations [20,21], raising concerns about generalizability to diverse populations.

To address this issue, we used the generator_yellow-stylegan2-config-f.pkl file, which was trained on 13,016 Asian facial images from the SeePrettyFace website [22]. We generated male and female digitally synthesized avatars with varying age progressions, ranging from 5 to 85 years old in increments of 10 years, to simulate realistic age-related facial changes.

### Generation and Comparison of FDface and DMface Fusion Images

Accurate generation of lesion positions and morphologies is critical for medical image generation [23]. While generative adversarial networks (GANs) excel at capturing global features, they often struggle with detailed pathological structures, as suggested by Kazeminia et al [24], and Han et al [25]. To mitigate this, we used Google’s MediaPipe library [26] for facial landmark detection. The Face Mesh detector identifies 3D coordinates for 468 facial landmarks (see Multimedia Appendix 3).

Affine transformations preserve collinearity and parallelism [26], making them suitable for adjusting discrepancies in the eye regions between patient and avatar images. We applied the Open Computer Vision Library (OpenCV) warpAffine() function [27] to adjust the eye regions while preserving the original proportions and positions. These transformed images, referred to as preliminary fusion images (see Figure 1), were then used for the fusion process.

**Figure 2.**
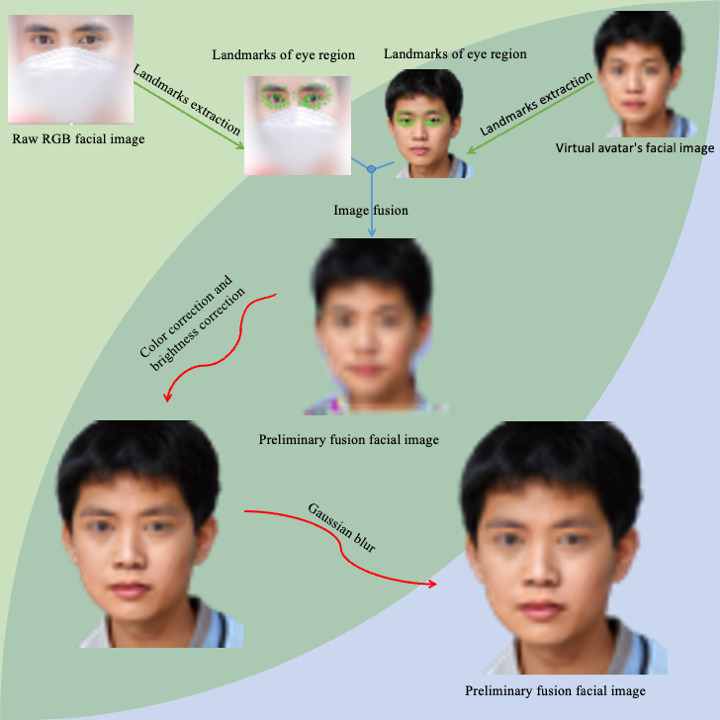
Workflow for the image fusion process. RGB: red, green, and blue.

During image fusion, disparities in skin tone, brightness, and unnatural fusion boundaries can arise, potentially affecting diagnostic accuracy. To rectify this, we applied RGB color correction [17,18] to harmonize patient images with avatar facial features. In addition, a Gaussian blur [28-30] was applied to the fused image edges to smooth boundary transitions [31] and create a more natural appearance. Figure 2 shows the detailed process by which the final high-quality fusion images (referred to as FDface) are generated through the fusion of raw RGB facial images and digitally synthesized avatar facial images. The workflow begins with the extraction of landmarks from both the raw images and the digitally synthesized avatars, followed by the image fusion step. This is followed by color correction and background adjustment to ensure consistency and improve visual appeal. Finally, Gaussian filtering is applied to smooth the image, further enhancing its quality and resulting in the final high-quality fused facial images. Furthermore, we generated both DMface and FDface images by applying the DM technique and Digital FaceDefender to the same original images for comparative analysis.

### Reidentification Risk Assessment

To assess the effectiveness of Digital FaceDefender in reducing reidentification risks while preserving essential facial features necessary for accurate diagnoses, we conducted a comparative analysis against DM using the Detailed Expression Capture and Animation (DECA) [8] framework. Specifically, we used the FLAME model to reconstruct facial images, generating DMface and FDface representations from the CASIA-FaceV5 dataset, which comprises 2500 facial images from 500 individuals, each captured in five different poses. These reconstructed images were labeled as FLAME_DMface and FLAME_FDface, respectively.

To evaluate the reduction in reidentification risk associated with Digital FaceDefender, we used ArcFace (IR-SE50 model) to compute facial similarity scores and Rank-1 accuracy. Specifically, we separately quantified the mean similarity scores between FDface and FLAME_FDface images and their corresponding original facial images. These similarity scores indicate the extent to which the generated images resemble the original faces. In addition, we used Rank-1 accuracy to assess whether FDface and FLAME_FDface images could be reidentified as their original counterparts using ArcFace.

### Statistical Analysis for Diagnostic Benchmark Establishment

To determine the sample size required for constructing the diagnostic benchmark, we first conducted a pilot study with $n_{pilot}=50$ to estimate the SD ($\sigma_{pilot}$) of the difference in pixel distances along the x-axis between the left and right eyes. The margin of error was then calculated by using the formula ($E=Z_{\frac{\alpha }{2}}\times \frac{\sigma_{pilot}}{\sqrt{n_{pilot}}}=1.96\times \frac{3.31}{\sqrt{50}}=0.92$ , where $Z_{\frac{\alpha }{2}}=1.96$ correspond to a 95% confidence level). Based on these estimates, the final required sample size (n) was determined using the standard sample size formula:


$$n={\left(\frac{Z_{\frac{\alpha }{2}}\cdot \sigma_{\text{pilot}}}{E}\right)}^{2}={\left(\frac{1.96\cdot 3.63}{0.92}\right)}^{2}=59.81$$

Finally, a total of 1136 healthy individuals were included in our study, exceeding the estimated sample size (n=60) and ensuring sufficient statistical power for establishing the diagnostic benchmark.

**Figure 3.**
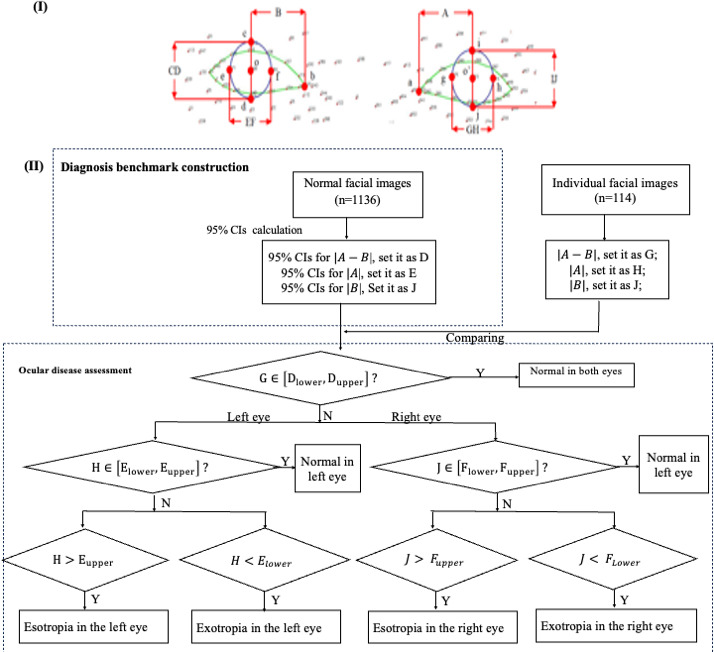
Measurement and comparison of pixel distances along the x-axis between the left and right eyes.

Pixel distances (|A|, |B|, |CD|, |EF|, |GH|, and |IJ|) along the x- and y-axes for both eyes were calculated between the iris center (o and o’) and reference points (a, b, c, d, e, f, g, h, i, j) in 1136 healthy individuals (see Figures 3II). The difference in pixel distances along the x-axis between the left and right eyes was denoted as |A-B|. Outlier detection was performed using the IQR method, excluding data points outside 1.5 times the IQR from the lower and upper quartiles. After outlier removal, 95% CIs for the pixel distances within the eye region were calculated. For variables that followed an approximately normal distribution (assessed by visual inspection and normality tests), we used the normal distribution method to calculate CIs. For variables that deviated from normality, we applied the bootstrap method to obtain more robust CI estimates. These diagnostic benchmarks were validated on an independent dataset of 114 individuals not included in the original sample. Inter-rater consistency between the diagnostic benchmarks and ophthalmologists’ assessments was evaluated using Cohen Kappa. The significance level was set to .05. Statistical analysis was performed using RStudio (version 1.1.463; Posit), which is developed and maintained by Posit.

The established diagnostic benchmarks were then used to identify ocular abnormalities, such as esotropia and exotropia (see Multimedia Appendix 4).

### Digital FaceDefender Platform

We developed the Digital FaceDefender platform to support ophthalmologists and researchers in their work, offering a user-friendly interface for privacy protection and auxiliary diagnostic assistance.

## Results

### Image Processing

Figure S1 in Multimedia Appendix 5 illustrates certain challenges due to low resolution (640×480), nonideal shooting angles, and intricate backgrounds, such as a blue curtain. These factors could hinder accurate representation of critical eye regions. As a representative example, we enhanced Figure S1A to a high-resolution version (2436×1944) shown in Figure S1B, both in Multimedia Appendix 5. Additional corrections included aligning the pupils horizontally in Figure S1C in Multimedia Appendix 5 and refining the cropping to emphasize the central facial region more clearly in Figure S1D in Multimedia Appendix 5.

### Creation of Digitally Synthesized Avatars

As depicted in Multimedia Appendix 6, we generated 2 sets of digitally synthesized avatars representing Asian males and females aged from 5 to 85 years at 10-year intervals. These avatars are used as templates in subsequent image fusion processes to ensure the preservation of critical facial features essential for medical diagnoses. Furthermore, a set of digitally synthesized avatars representing Caucasian individuals was also created.

### Generation and Comparison of FDface and DMface Fusion Images

Using Google’s MediaPipe library, we focus on 52 key landmarks within the eye and periocular regions, including the eyelids, eye sockets, irises, and brow arches, for both patients and avatars. These landmarks are used to define closed ROIs for subsequent image fusion. OpenCV is then applied to adjust the eye regions, resulting in transformed images, referred to as preliminary fusion images (see Figure 1), which are subsequently used in the fusion process.

**Figure 4.**
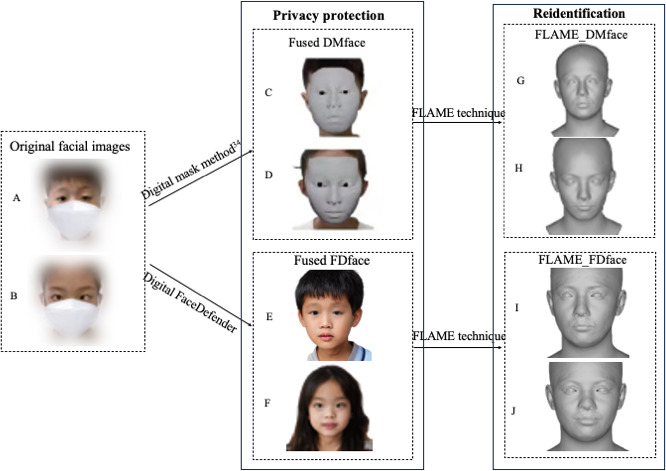
Comparison of privacy protection and reidentification risk: DM versus Digital FaceDefender (the 2 facial images are sourced from Yang et al [4]). DM: Digital Mask; FLAME: Faces Learned with an Articulated Model and Expressions.

Figure 4 presents the final fused images generated by both the DM method and our proposed Digital FaceDefender approach. The DMface images produced using the DM method are shown in Figure 4C and D, while the FDface images created by Digital FaceDefender are depicted in Figure 4E and F. The difference in fused images generated by DM and Digital FaceDefender highlights that Digital FaceDefender not only meets the privacy protection requirements but also preserves essential features related to ocular diseases. The resulting natural-looking fused images enhance clinician-patient empathy, which is crucial for accurate diagnosis and personalized treatment. Furthermore, the final fused images generated using the various ethnic digitally synthesized avatars, as presented in Multimedia Appendix 7, demonstrate the generalizability of the proposed Digital FaceDefender methodology.

### Reidentification Risk Assessment

Figure 4 illustrates the difference in reidentification risk between the DM method and the Digital FaceDefender approach. When applying the FLAME reconstruction technique to both DMface and FDface images, the FLAME_DMface images (Figure 4G and H) exhibit a higher reidentification risk compared to FLAME_FDface images (Figure 4I and J). This increased risk stems from the preservation of key facial contours, including the face shape, nose, eyes, and mouth, which enables effective reconstruction through FLAME in the DM method.

As shown in Table 1 and Multimedia Appendices 8 and 9, the mean similarity score between FDface and raw facial images is 0.31. While this value suggests that FDface retains some identifiable facial features, potentially beneficial for auxiliary diagnosis, it remains below the reidentification threshold established by leading facial recognition systems. For example, Microsoft’s Face API reports that similarity scores above 0.5 indicate a potential match [32]. The significantly lower mean similarity score of 0.09 for FLAME_FDface versus raw facial images suggests that reconstructing the original face from FDface images is highly challenging, confirming that Digital FaceDefender significantly reduces reidentification risk while enhancing privacy protection.

**Table 1.** Evaluating the mean similarity between FDface and FLAME_FDface images and raw facial images.

Comparison	Mean similarity
FDface versus raw facial images	0.31
FLAME_FDface versus raw facial images	0.09

Further validation comes from Rank-1 accuracy analysis across different facial poses (see Tables2  3). Notably, for Pose #2 and Pose #3 (see Table 2) and Poses #2-#5 (see Table 3), the Rank-1 accuracy is 0, indicating that no successful reidentifications occurred in these conditions. Even for Pose #4 (0.02) and Pose #5 (0.04), the Rank-1 accuracy remains near zero, confirming that Digital FaceDefender effectively mitigates reidentification risks across various facial angles.

These results underscore the robustness of our proposed Digital FaceDefender in balancing privacy protection with retention of diagnostic features.

**Table 2.** Evaluating the Rank-1 accuracy between FDface images and raw facial images with different poses.

Pose	Rank-1 accuracy
Pose #2	0
Pose #3	0
Pose #4	0.02
Pose #5	0.04

**Table 3.** Evaluating the Rank-1 accuracy between FLAME_FDface images and raw facial images with different poses.

Pose #	Rank-1 accuracy
Pose #2	0
Pose #3	0
Pose #4	0
Pose #5	0

### Diagnostic Benchmark Establishment

**Figure 5.**
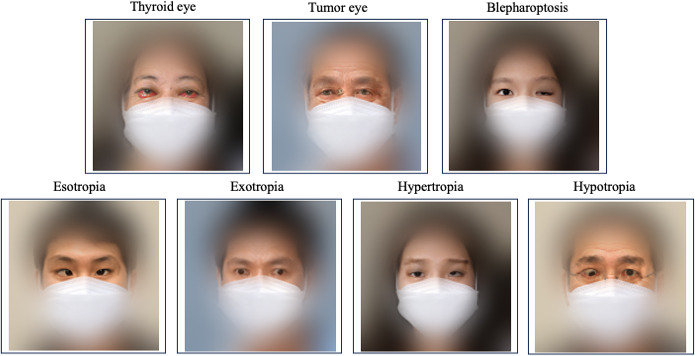
Clinical diagnoses of ocular diseases based on fused images.

Using the developed diagnostic benchmarks, we evaluated their agreement with ophthalmologists’ assessments for ocular disease detection (see Figure 5). As shown in Table 4, Cohen Kappa analysis demonstrated a fair agreement for the left eye (κ=0.37) and a moderate agreement for the right eye (κ=0.445). Both agreements were statistically significant (*P*<.001), indicating the reliability of the benchmarks. However, factors such as head posture and image quality may influence diagnostic accuracy.

**Table 4.** Cohen Kappa analysis in classifying right and left eye strabismus.

	Kappa	*P* value
Left eye	0.37	<.001
Right eye	0.45	<.001

### Digital FaceDefender Platform

The Digital FaceDefender platform is available as a website [33]. Multimedia Appendix 10, along with the accompanying Multimedia Appendix 11, demonstrates the effectiveness of the Digital FaceDefender Platform in protecting patients’ facial privacy while simultaneously supporting the diagnosis of ocular diseases. In addition, the Digital FaceDefender platform utilizes the developed diagnostic benchmarks to identify raw images with suboptimal camera angles, providing text prompts to flag them as poor-quality images.

## Discussion

This study introduces Digital FaceDefender to safeguard patients’ privacy and achieve auxiliary diagnosis. Collectively, the comparison of DMface images, FDface images, FLAME_DMface images, and FLAME_FDface images, along with the evaluation metrics including Rank-1 accuracy, mean similarity, and Cohen Kappa, demonstrates that Digital FaceDefender effectively facilitates auxiliary diagnosis while concurrently safeguarding patient privacy.

Quantitative analysis, including mean similarity scores and Rank-1 accuracy, demonstrates that Digital FaceDefender substantially reduces reidentification risks while effectively preserving critical diagnostic features. In contrast, existing anonymization methods exhibit certain limitations. For example, Shawn Shan et al [34] introduces subtle perturbations to facial images to prevent unauthorized facial recognition. However, these perturbations can be removed using super-resolution models, adversarial training, or denoising techniques, thereby restoring identifiable facial features and significantly weakening privacy protection. Similarly, FaceObfuscato [35] disrupts reidentification attacks, making the method resistant to adversarial optimization techniques. However, it remains vulnerable to non–gradient-based and adaptive attacks that leverage auxiliary information or brute-force reconstruction. In addition, its transformations may distort critical eye-region details, potentially degrading performance in ocular auxiliary diagnosis. Face Deidentification [36] uses GAN-based face synthesis to generate realistic, high-resolution anonymized faces. While this method enhances image fidelity, it can be susceptible to GAN inversion attacks. In contrast, Digital FaceDefender achieves lower reidentification and similarity scores, demonstrating superior robustness in privacy protection. Unlike diffusion-based anonymization methods, such as the approach proposed by Kung et al [37], which suffer from uncontrollable anonymization levels due to the nonlinear latent space, Digital FaceDefender enables precise control over the strength of anonymization, ensuring that key ocular features remain intact for auxiliary diagnosis. Furthermore, diffusion-based anonymization can degrade expression recognition accuracy, whereas Digital FaceDefender generates visually natural and diagnostically meaningful fused images. Compared to DeepPrivacy [38],which uses a StyleGAN-like architecture to synthesize high-resolution, photorealistic faces. However, it remains vulnerable to adversarial attacks. Digital FaceDefender preserves clinically relevant facial features while minimizing reidentification risks.

However, there are several limitations to this study. First, while Google’s MediaPipe can detect 468 facial landmarks, this number may be insufficient to accurately represent the ROI, particularly since only 16 landmarks are assigned to the eyelids and 5 to the iris for each eye. Second, MediaPipe faces challenges in accurately localizing the ROI, especially when image quality is compromised. Third, aligning pixel distances in the ROI on the digitally synthesized avatar’s face with the actual ROI size on the patient’s face remains problematic. A possible solution could involve using a horizontal ruler during photography as a reference for scaling. Fourth, the quality of image fusion is influenced by factors such as camera equipment, shooting angles, and environmental conditions, highlighting the need for standardized imaging protocols. Fifth, enhancing the accuracy of fusion boundaries will require larger and more diverse training datasets, which remain scarce, particularly for rare medical conditions. Finally, we explore some novel advanced techniques, like Hugging Face using Stable Diffusion (V3.5), developed by Hugging Face [35], to generate digitally synthesized avatars from various ethnic backgrounds. However, the results produced by Stable Diffusion did not surpass those generated by StyleGAN2 in terms of fusion quality (see Multimedia Appendix 7). The reason for this could be the significantly higher resolution of virtual avatars produced by Stable Diffusion compared to the raw facial images, which may have introduced challenges in achieving seamless fusion effects (see Multimedia Appendix 7).

In conclusion, unlike other privacy protection technologies, Digital FaceDefender demonstrates dual efficacy: it preserves privacy while facilitating auxiliary diagnoses. Although our preliminary findings are promising, further refinement of the image fusion process is essential to enhance the realism and accuracy of the resultant images. In addition, improving the automatic matching between the virtual avatar’s face and the patient’s face has the potential to reduce the workload of health care professionals. Future research should focus on these areas to advance the utility and applicability of Digital FaceDefender in clinical practice.

## Supplementary material

10.2196/66873 Multimedia Appendix 1 Images from “A digital mask to safeguard patient privacy.”

10.2196/66873 Multimedia Appendix 2 Ethical approval.

10.2196/66873 Multimedia Appendix 3 The landmarks of face and periocular region.

10.2196/66873 Multimedia Appendix 4 The detection of blepharoptosis in the right eye and left eye.

10.2196/66873 Multimedia Appendix 5 Image preprocessing for images with low-quality.

10.2196/66873 Multimedia Appendix 6 Digitally synthesized avatars of Asian males and females representing various age groups.

10.2196/66873 Multimedia Appendix 7 The final fused images with various ethinic digital avatars.

10.2196/66873 Multimedia Appendix 8 Comparison of similarity rates between FDface and original facial images.

10.2196/66873 Multimedia Appendix 9 Comparison of similarity rates between FLAME_FDface and original facial images.

10.2196/66873 Multimedia Appendix 10 Illustration of the Digital FaceDefender platform.

10.2196/66873 Multimedia Appendix 11 Implementation video of the Digital FaceDfender platform.

## Abbreviations

AI: artificial intelligence
DECA: Detailed Expression Capture and Animation
DM: Digital Mask
FFHQ: Flickr-Faces-HQ
FLAME: Faces Learned with an Articulated Model and Expressions
GAN: generative adversarial network
RGB: red, green, and blue
ROI: region of interest
