# Correction: Artificial Intelligence–Enabled Facial Privacy Protection for Ocular Diagnosis: Development and Validation Study

**DOI:** 10.2196/84928

**Published:** 2025-12-04

**Authors:** Haizhu Tan, Hongyu Chen, Zhenmao Wang, Mingguang He, Chiyu Wei, Lei Sun, Xueqin Wang, Danli Shi, Chengcheng Huang, Anping Guo

**Affiliations:** 1 Department of Preventive Medicine, Shantou University Medical College, 22 Xinling Rd, Shantou, 515031, China, 86 13318055534; 2 Department of Optoelectronic Information Science and Engineering, Physical and Materials Science College, Guangzhou University, Guangzhou, China; 3 Han’s Laser Technology Industry Group Co., Ltd, Shenzhen, China; 4 Joint Shantou International Eye Center of Shantou University and The Chinese University of Hong Kong, Shantou, China (Hong Kong); 5 The Hong Kong Polytechnic University, Kowloon, Hong Kong, China (Hong Kong); 6 Department of Ophthalmology, the Fourth Affiliated Hospital of Harbin Medical University, Harbin, China; 7 University of Science and Technology of China, Hefei, China; 8 Department of Pharmacy, First Affiliated Hospital of University of Science and Technology of China, Division of Life Sciences and Medicine, University of Science and Technology of China, Hefei, China

In “Artificial Intelligence–Enabled Facial Privacy Protection for Ocular Diagnosis: Development and Validation Study” [1], the authors noted several errors.

Co-first authorship has been noted within the “Acknowledgements” section as follows:

HC, ZW, and MH are co-first authors on this work.

In affiliation 5, the institution was changed from:

The Hong Kong Polytechnic University

To read:

The Hong Kong Polytechnic University, Kowloon

In addition, affiliation 6 has been changed from:

Department of Ophthalmology, the Fourth Affiliated Hospital of Harbin Medical University, Haerbin, China

To read:

Department of Ophthalmology, the Fourth Affiliated Hospital of Harbin Medical University, Harbin, China

The corrections will appear in the online version of the paper on the JMIR Publications website, together with the publication of this correction notice. Because this was made after submission to PubMed, PubMed Central, and other full-text repositories, the corrected article has also been resubmitted to those repositories.
