# Supplementary figures and images for: Artificial Intelligence–Enabled Facial Privacy Protection for Ocular Diagnosis: Development and Validation Study
Source: J Med Internet Res. 2025 Jul 9;27:e66873. doi: 10.2196/66873 (PMC12266301; doi:10.2196/66873)

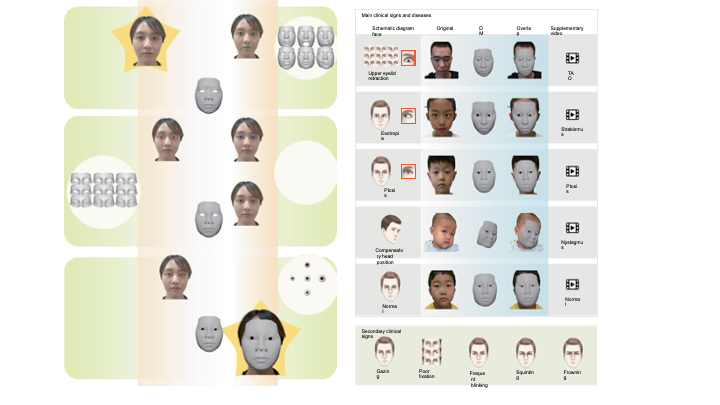

Supplement: Multimedia Appendix 1 [file jmir-v27-e66873-s001.png]

1. The Landmarks of face and periocular region


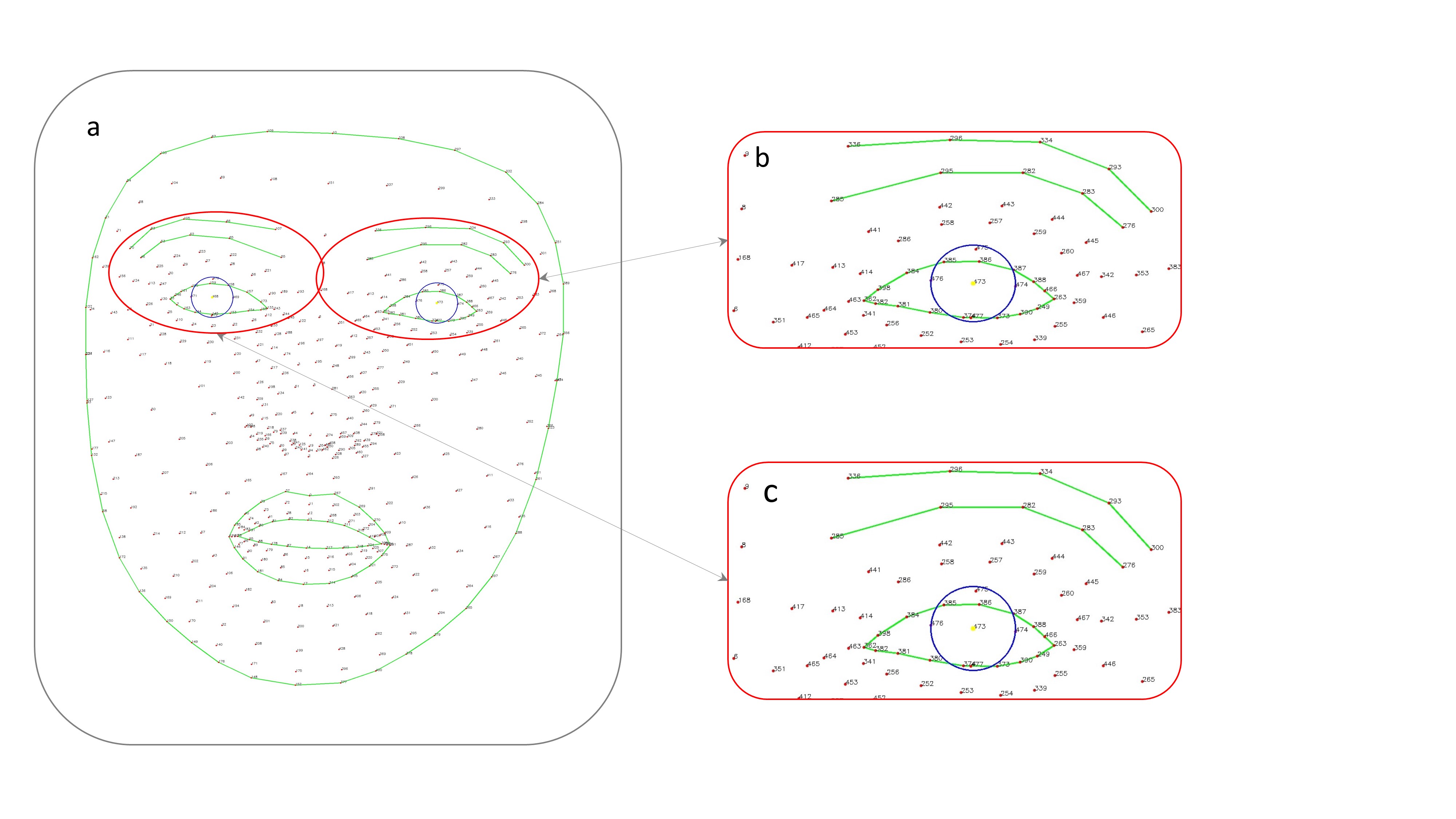

Supplement: Multimedia Appendix 3 [file jmir-v27-e66873-s003.docx]

6. The detection of blepharoptosis in the right eye and left eye


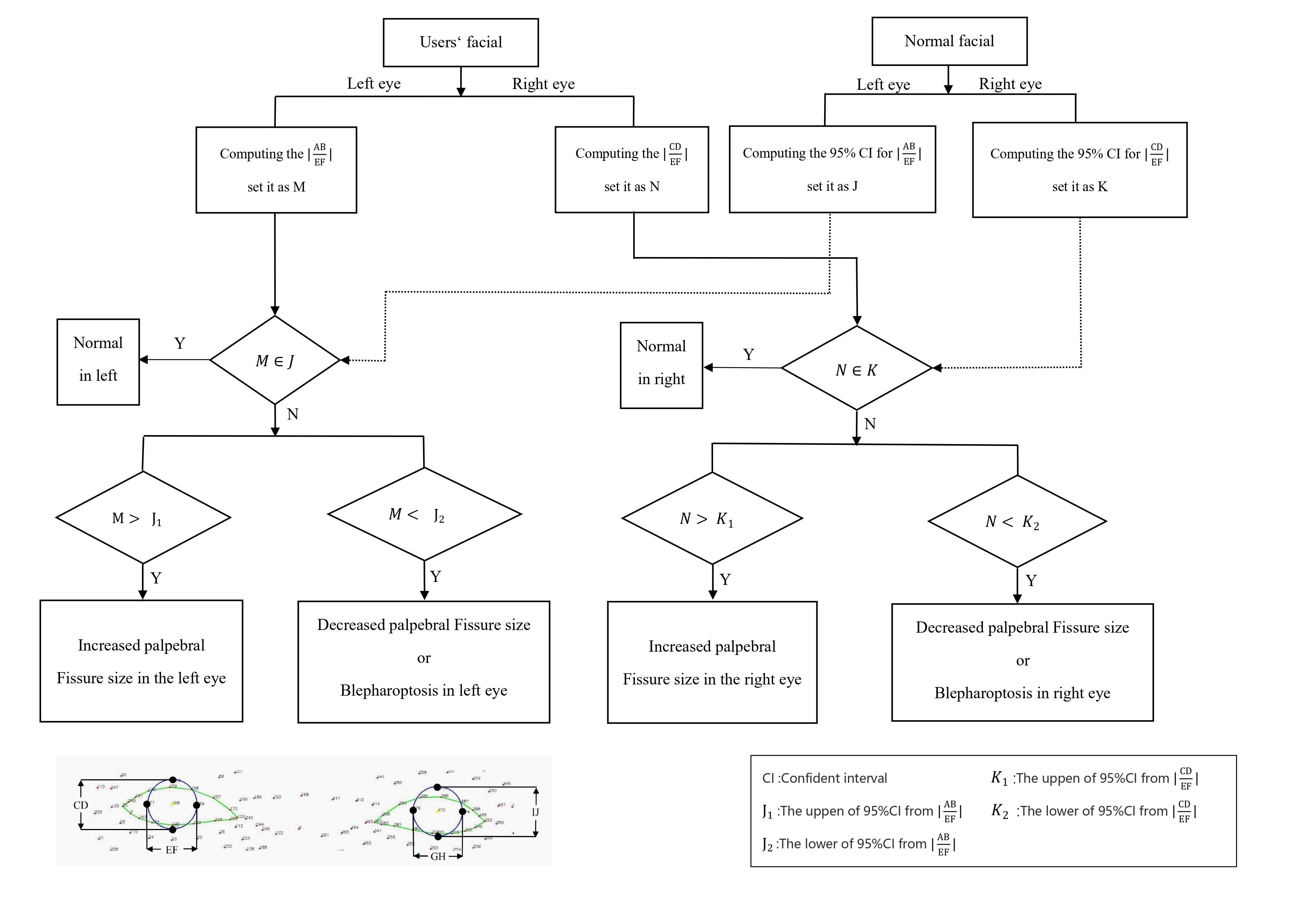

Supplement: Multimedia Appendix 4 [file jmir-v27-e66873-s004.docx]

1. Virtual avatars of Asian males and females representing various age groups


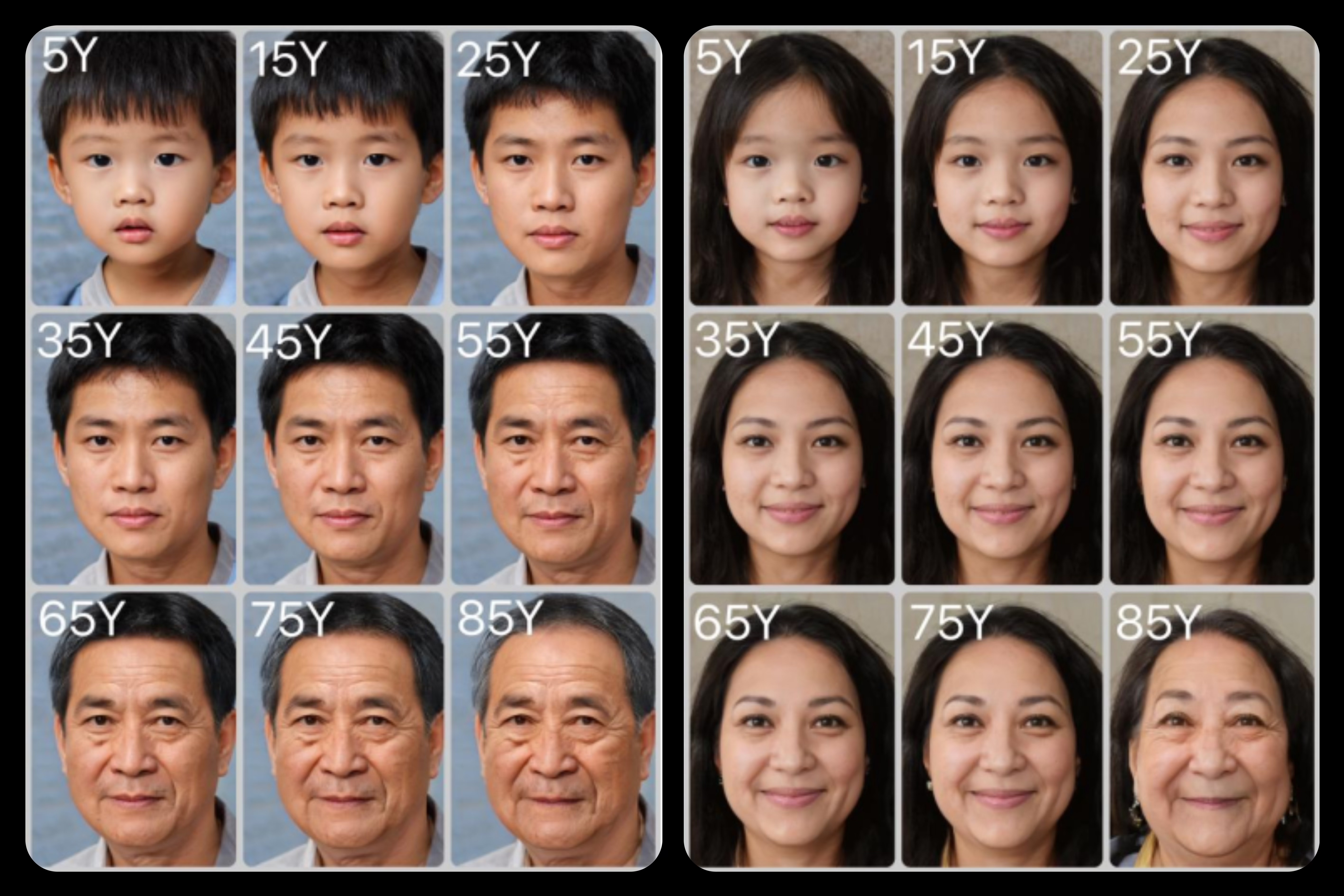

Supplement: Multimedia Appendix 6 [file jmir-v27-e66873-s006.docx]

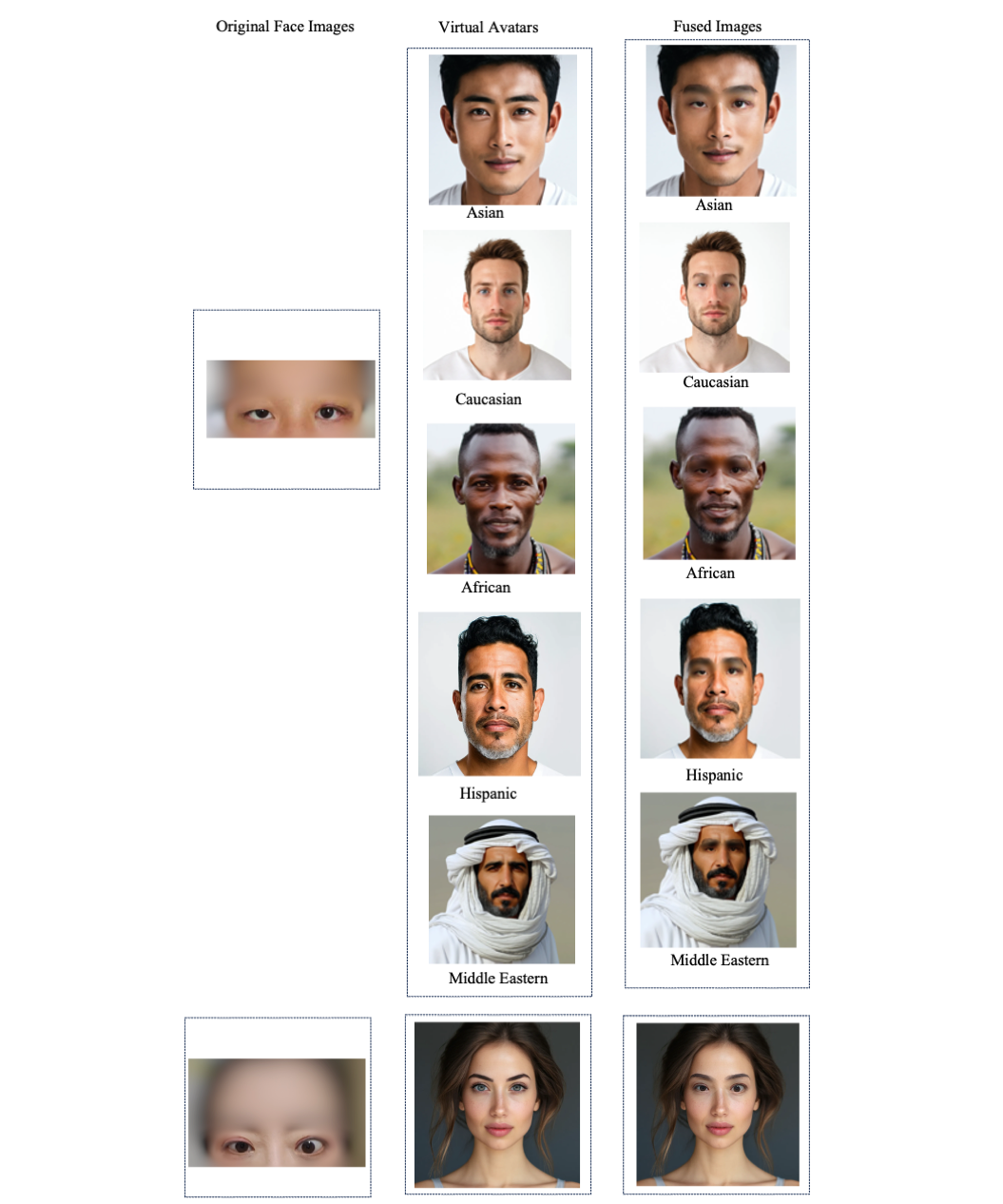

Supplement: Multimedia Appendix 7 [file jmir-v27-e66873-s007.png]
