# Supplementary material for: Artificial Intelligence–Enabled Facial Privacy Protection for Ocular Diagnosis: Development and Validation Study
Source: J Med Internet Res. 2025 Jul 9;27:e66873. doi: 10.2196/66873 (PMC12266301; doi:10.2196/66873)
Supplement: Multimedia Appendix 2 [file jmir-v27-e66873-s002.pdf]

哈尔滨医科大学附属第四医院医学伦理审查批件

编号：2023-伦理审查-54

**项目名称**	利用眼底彩照和眼底荧光造影图片以及眼科病人脸部照片进行缺血性心脏病辅助诊断和隐私保护以及眼周疾病辅助诊断		
**研究申办方**	哈尔滨医科大学附属第四医院		
**主要研究者单位**	哈尔滨医科大学附属第四医院		
**主要研究者**	孙蕾	**审查形式**	会议审查
**日　期**	2023 年 12 月 20 日		
**主任委员**	郭松	**副主任委员**	朱丽影
**参会委员**	应到 17 人，实到 12 人，其中法学专家、伦理学专家、社会人士均出席。		
**审查资料**	1.医学伦理审查项目申请表<br>2.研究方案（版本号 1.0 版本日期 2023 年 9 月 1 日）<br>3.知情同意书（版本号 1.0 版本日期 2023 年 9 月 1 日）<br>4.主要研究者声明<br>5.科研承诺书		

主要研究者资格评价：

主要研究者从事相关工作多年，具备严谨的科学态度和扎实的科研能力，有承担科研项目并组织开展的经验，具备完成该项目的能力。

设计方案评价：

研究具有科学数据支撑，具备科学性。研究方案设计明确，技术路线清晰可行，具备可行性。研究纳入排除标准恰当，风险可控，具备合理性。

知情同意评价：

知情同意书告知要素齐全，对研究参与者参加研究的风险和受益做到了充分描述，语言整体通俗易懂，能够达到研究参与者的认知标准，符合伦理要求。

特别声明：

本次审查有效期为批件签署日期后的 12 个月，申请人应注意所有材料未经医学伦理委员会审核不得擅自修改，每年应递交年度进展报告。

如在项目实施期有任何偏离或违背方案、严重不良事件、暂停或终止项目等，应及时向医学伦理委员会递交相关材料。项目完成后应向医学伦理委员会递交完成报告。

**审查意见：**<br>经会议审查和委员表决，最终结果为同意 12 票，修改后同意 0 票，不同意 0 票。<br>**最终审查结果为通过伦理审查，同意开展。**	**哈尔滨医科大学附属第四医院医学伦理委员会**<br>**（签字盖章生效）**<br>**2024 年 1 月 2 日**
