# Supplementary material for: Artificial Intelligence–Enabled Facial Privacy Protection for Ocular Diagnosis: Development and Validation Study
Source: J Med Internet Res. 2025 Jul 9;27:e66873. doi: 10.2196/66873 (PMC12266301; doi:10.2196/66873)
Supplement: Multimedia Appendix 5 [file jmir-v27-e66873-s005.docx]

Figure S1．Image Preprocessing for images with low-quality


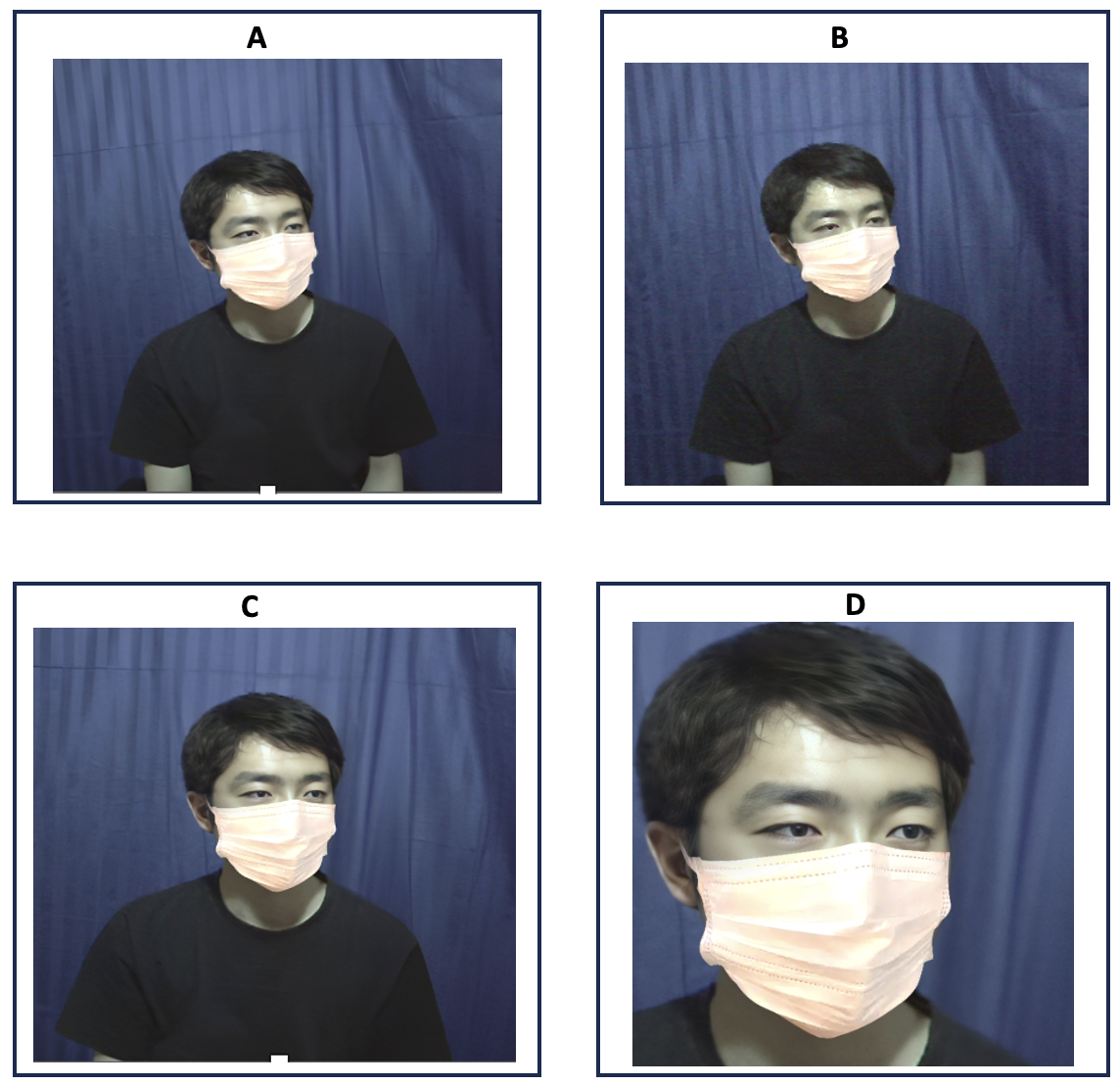


(Note:

A: yellow-face image with RGB, B: images with higher resolution,

C: the pupils and both eyes are adjusted on the same horizontal line,

D：cropped image)
