# Supplementary material for: Artificial Intelligence–Enabled Facial Privacy Protection for Ocular Diagnosis: Development and Validation Study
Source: J Med Internet Res. 2025 Jul 9;27:e66873. doi: 10.2196/66873 (PMC12266301; doi:10.2196/66873)
Supplement: Multimedia Appendix 9 [file jmir-v27-e66873-s009.docx]

Comparison of Similarity Rates between FDface Images and Original Facial Images
