# Supplementary material for: Artificial Intelligence–Enabled Facial Privacy Protection for Ocular Diagnosis: Development and Validation Study
Source: J Med Internet Res. 2025 Jul 9;27:e66873. doi: 10.2196/66873 (PMC12266301; doi:10.2196/66873)
Supplement: Multimedia Appendix 10 [file jmir-v27-e66873-s010.docx]

7. Illustration of the Digital FD Platform

**
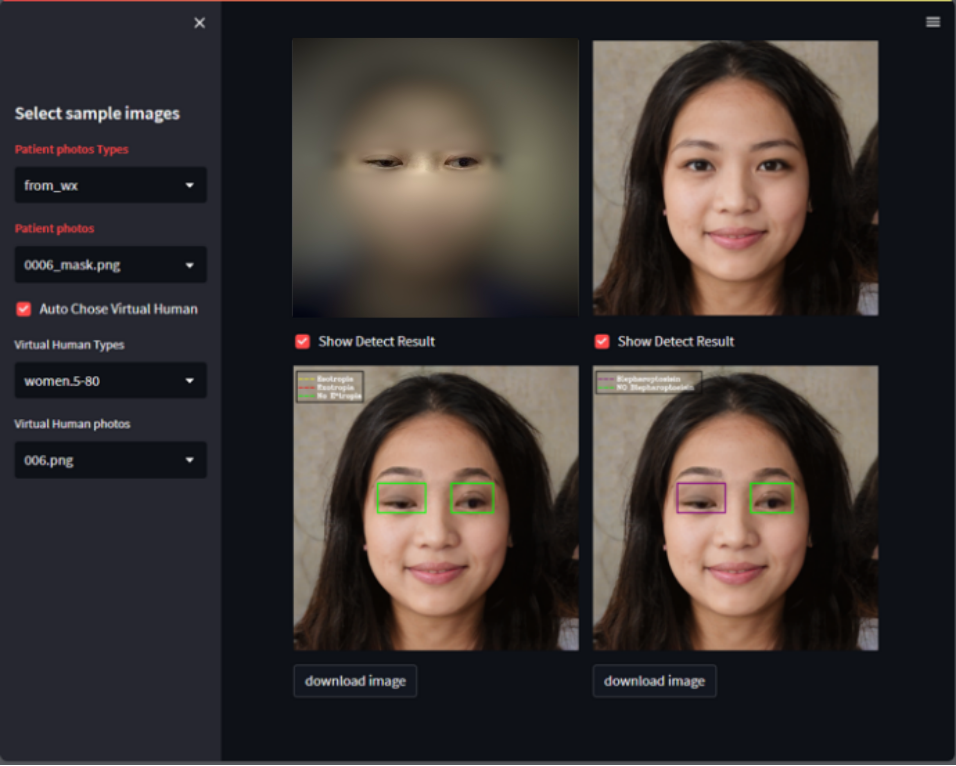
**

Note: As depicted in the upper-left image of Figure 8, facial obfuscation has been applied to authentic human facial images using Google’s Snapseed software (https://chrome.google.com/webstore/detail/snapseed-online-pc-photo/fnmgbgbidhadiadgcnfmamimcepijdkm) to maintain the highest level of patient privacy protection when showcasing screenshots to illustrate the effectiveness of the Digital FD Platform. The Upper-right image depicted the final fusion image. Main clinical signs for diagnosis of Exotropia /Esotropia(bottom left) and blepharoptosis (bottom right) studied were distinguished through a frame using different colors. )
